# Central and peripheral refraction measured by a novel double-pass instrument

**DOI:** 10.1364/BOE.489881

**Published:** 2023-05-10

**Authors:** Dimitrios Christaras, Spyridon Tsoukalas, Petros Papadogiannis, Charlie Börjeson, Moa Volny, Linda Lundström, Pablo Artal, Harilaos Ginis

**Affiliations:** 1 Diestia Systems, 77-79 Platonos str., 14401, Athens, Greece; 2 Department of Research, Athens Eye Hospital, 44 Vouliagmenis Av., 17631, Glyfada, Greece; 3 Department of Applied Physics, Royal Institute of Technology, Stockholm, 11421, Sweden; 4 Laboratorio de Optica, Universidad de Murcia, Campus de Espinardo (Edificio 34), E-30100 Murcia, Spain

## Abstract

A novel double-pass instrument and its data analysis method for the measurement of central and peripheral refraction is presented and validated in a group of healthy subjects. The instrument acquires in-vivo, non-cycloplegic, double-pass, through-focus images of the eye’s central and peripheral point-spread function (PSF) using an infrared laser source, a tunable lens and a CMOS camera. The through-focus images were analyzed to determine defocus and astigmatism at 0° and 30° visual field. These values were compared to those obtained with a lab-based Hartmann-Shack wavefront sensor. The two instruments provided data showing good correlation at both eccentricities, particularly in the estimation of defocus.

## 1. Introduction

The quality of our central vision plays a crucial role in our ability to perform complex tasks, such as recognizing faces or reading. However, peripheral vision is also important for detecting stimuli and orienting the eye towards them, as well as for detecting movement and being aware of our surroundings. While a decrease in central vision is easily noticed by the patient, a reduction in peripheral vision may go unnoticed. Studies have shown that an increase in refractive errors in the peripheral field of vision can lead to a significant decrease in the detection of stimuli [1], which can have serious consequences in tasks such as walking, where obstacles such as stair steps can be missed, or while driving, where awareness of other vehicles and the surroundings is crucial [2]. Currently, peripheral refraction was done either using custom laboratory setups or adapted open-view autorefractors designed for the assessment of central refraction [3], but there has not been a commercial clinical instrument focusing on the quick and easy assessment of peripheral refraction. The purpose of this work is to demonstrate a novel double-pass instrument for the assessment of peripheral image quality, which could, in the future, reach clinical practice.

The assessment of peripheral refraction is also important in myopia research since peripheral image quality is believed to have a role in reducing the rate of myopia progression with optical control interventions. The main hypothesis is that the eye growth may depend on peripheral defocus; it is believed that a more myopic periphery will result in reduced eye growth for a myopic eye [4]. However, there is not yet enough conclusive evidence to firmly establish the relationship between peripheral defocus, astigmatism, and myopia.

Another important issue related to peripheral image quality is the increasing prevalence of cataract surgery [5], especially among younger patients due to the ease of the procedure, often as an alternative to refractive surgery [6]. Intraocular lenses (IOLs) used in cataract surgery have been primarily designed as thin lenses to simplify surgical technique, resulting in increased peripheral astigmatism compared to the natural crystalline lens of the eye [7]. A recent study has shown that standard monofocal IOLs, commonly used in cataract surgery, can cause a decrease in the detection threshold in the far periphery [8] and the use of IOLs designed to improve peripheral image quality can increase peripheral field sensitivity [9]. Additionally, recent research shows a significant field distortion in the pseudophakic eye [10]. These phenomena could affect the functional vision of the pseudophakic patient not only in demanding tasks such as driving [11–13], but in everyday tasks such as walking [14], crossing a busy street, or participating in sports.

In this work we present a new double-pass instrument, using a novel algorithm for the in-vivo, quick, and accurate measurement of the central and peripheral refraction of the human eye. The instrument was compared with a Hartmann-Shack (HS) custom-made aberrometer developed for laboratory use.

## 2. Methods

### 2.1 Double pass prototype instrument

**Fig. 1.**
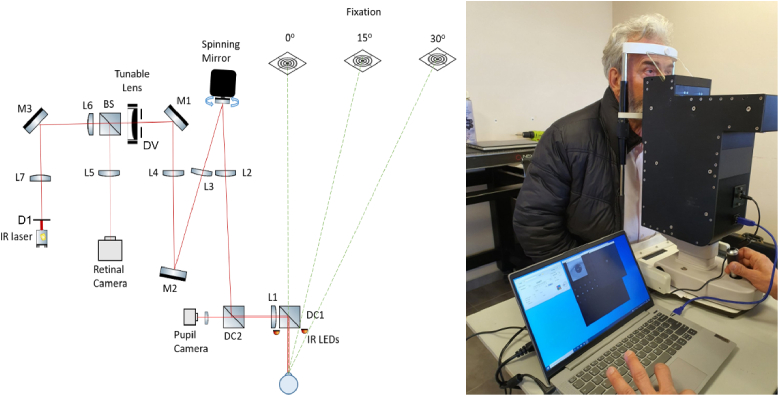
Schematic of the instrument (left) and photo of the instrument measuring a subject (right).

The double-pass (DP) instrument consists of an infrared laser (780 nm) that produces a retinal point-like spot, a focus tunable lens capable of inducing −10 D to +10 D of defocus, and a CMOS camera for capturing the spot's image. A schematic of the instrument is shown in Fig. 1.

The laser source produces a collimated beam of 2 mm diameter. A 1:1 relay telescope (L7, L6) is used to conjugate the 2 mm aperture D1 (first pass) on the variable aperture DV, which can produce apertures of diameters from 2.5 mm to 7 mm with a step of 0.5 mm for the second pass. Subsequently, the aperture is conjugated on a spinning mirror through a 1:1 relay telescope (L4, L3) and on the eye’s pupil through another 1:1 relay telescope (L1, L2). The relay optics project the laser point source on the retina through a 2 mm pupil. The spinning mirror scans a circular pattern of about 0.5° visual angle at roughly 6000 rpm on the retina and is used to minimize speckle from the coherent light source and to average a larger area on the retina to avoid localized non-uniformities, in the same fashion done by Hofer at al [15]. Light reflected from the retina follows the opposite path and it is projected onto a CMOS sensor through a beamsplitter (BS). The beam is “de-scanned” on its second pass from the spinning mirror and, therefore, the CMOS sensor captures a point source consisting of the sum of the light coming from the retinal circle. The exposure time of the camera and the angular speed of the spinning mirror are set such that a whole circle was projected on the retina during the exposure time of a single image. The tunable lens can rapidly cause the beam to converge or diverge both on the first pass from the laser source on the retina and the second pass from the retina on the CMOS sensor. The tunable lens has been proven to be a good alternative to the slow, bulky and expensive motorized Badal systems to adjust the focus; double pass systems using a tunable lens to record through focus images of the central double pass PSF have been also developed in the past [16].

The subject’s eye is aligned using 950 nm infrared illumination on the pupil and a second CMOS camera of lower resolution and the appropriate cold mirror allowing only wavelengths over 850 nm to transmit. The instrument follows the ISO15004-2:2007 on ophthalmic safety and the power of the laser on the retina is more than an order of magnitude below the Maximum Permissible Exposure. The entire instrument can rotate, while the subject maintains fixation at a far target (∼3 m) in an open-view configuration, to measure not only central, but peripheral refraction as well. An image of the instrument while measuring a subject in a laboratory setting can be seen in Fig. 1. In this work, all measurements were done at 3.5 mm pupil size in the second pass. In this experiment two measurement angles are chosen: central (0°) and peripheral (30°).

**Fig. 2.**
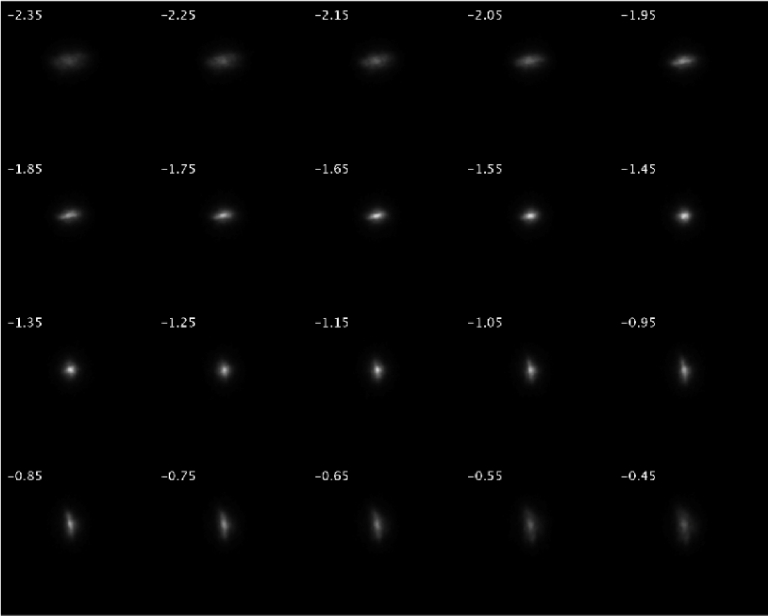
A sequence of through-focus images with the subject fixating at 0° (centrally).

To establish best focus, a series of through-focus images are recorded over a 20 D range with a step size of 0.1 D, and the image with the highest peak intensity value was selected. An example of a sequence of through-focus images acquired at 0° of visual angle can be seen in Fig. 2.

**Fig. 3.**
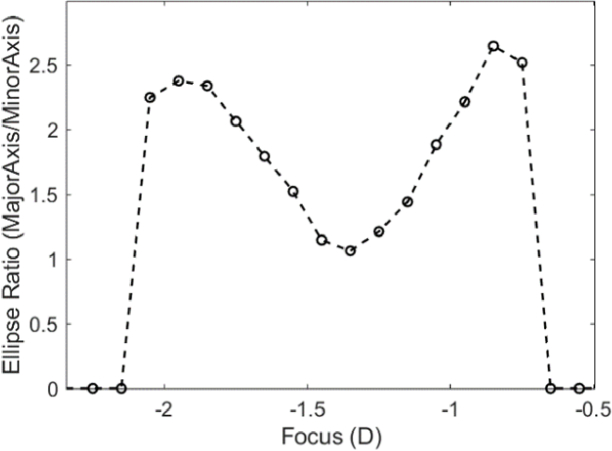
Astigmatism calculation from a set of through focus images. An ellipse shape is fitted on each PSF of Fig. 3, after a threshold is applied, and the ratio of the major to minor axis is calculated for each fitted ellipse (circles in the plot). The result is shown in the plot where the ratio at each defocus setting is shown.

Astigmatism was calculated by thresholding each image to get a solid oval shape and fitting an ellipse to determine the ratio of the two axes. The axis of astigmatism was defined as the axis of the image with the highest defocus value (negative cylinder notation) and its magnitude was defined as the dioptric difference between the two PSF images with the highest ratio. In the presence of astigmatism, best focus was determined as the mean of the dioptric values of these two elliptical images. An example of such a calculation is presented in Fig. 3, where the ellipse axis ratio at each defocus setting is shown.

In the example above, the astigmatism was found to be $R_{max1}-R_{max2}=-1.1\;\text{D}$ and sphere was $(R_{max1}+R_{max2})/2=-1.4\;\text{D}$ . The calculated astigmatism was verified by correcting it with the appropriate cylindrical trial lens (-1.5 D), following the method described by Guirao and Artal [17].

### 2.2. Hartmann-Shack aberrometer

The HS aberrometer used in this study is a custom-made laboratory prototype that can measure wavefront aberrations at 0° (central) and 30° (peripheral) of visual angle. The device is composed of two measurement channels, each equipped with a HS wavefront sensor that allows for the simultaneous acquisition of data from the two retinal sites. Further details on the device and the algorithm used to extract the Zernike coefficients, can be found in [18]. In this study participants were presented with an illuminated target displaying fine details (with angular frequencies up to 50 cycles per degree) while the lighting in the experimental room was reduced to allow for a larger pupil size. The target (a back-illuminated Maltese cross) was located about 3 m away. Each measurement lasted a few seconds and three repetitions were performed for the right eye of the participants. The Zernike coefficients (2^nd^, 4^th^ and 6^th^ orders) describing defocus and astigmatism were calculated for each subject at each eccentricity. A pupil of 3.5 mm was used for the analysis. Both instruments were located side-by-side so that fixation target, light conditions, and target distance remained the same for all measurements. The position of the subject was realigned between each repetition for both instruments.

### 2.3. Subjects

Fifteen healthy volunteers participated in the study. They were measured three times each, both centrally (0°) and peripherally at 30° in the nasal visual field, using the DP instrument as well as the HS aberrometer. Only the right eye of each patient was measured. Informed consent was obtained from all participants and the measurements were approved by the Swedish Ethical Review Authority, follow the tenants of Helsinki, and adhere to the European GDP Regulation.

### 2.4. Data analysis

The mean values for defocus and astigmatism for each subject were extracted at each eccentricity (center and periphery). Repeatability of the DP instrument was tested using the Bland-Altman analysis [19] for two consecutive measurements. For this analysis, the difference and the mean of the two consecutive measurements were plotted; the mean difference (bias) was also calculated as well as the upper and lower limits of agreement (LoA) following the formula upper LoA = mean + 1.96 standard deviation and lower LoA = mean – 1.96 standard deviation. For the comparison between the two different instruments, first Pearson’s correlation was calculated to evaluate the strength of the linear association between the measurements from each instrument. Agreement between the DP and the HS instruments was also investigated using Bland-Altman analysis. A p-value below 0.05 was needed to reject the null hypothesis and determine statistical significance.

## 3. Results

### 3.1 DP instrument repeatability

**Fig. 4.**
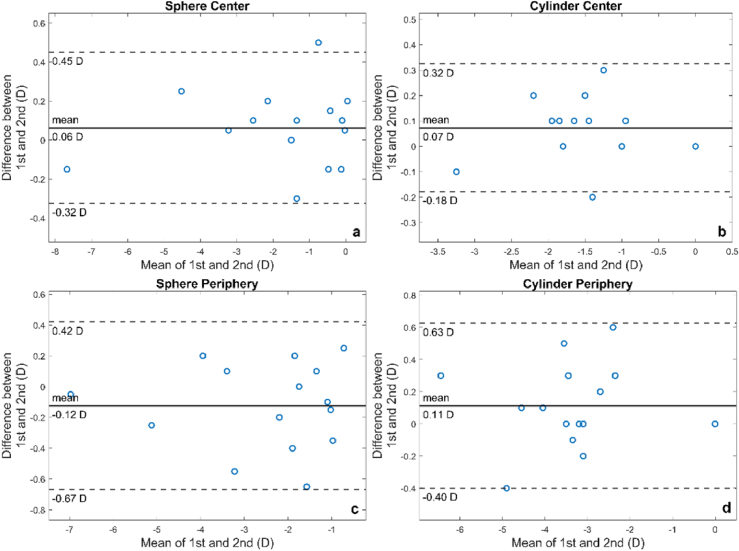
Bland-Altman plot for 1^st^ and 2^nd^ measurement for 15 subjects. Mean difference (bias) and limits of agreement are also shown in the plot.

The repeatability of the DP instrument was investigated using the Bland-Altman analysis, where the mean and the difference of the first and second measurement are calculated and plotted in Fig. 4(a)-(d) for central sphere, central astigmatism, peripheral sphere and peripheral astigmatism. The mean difference (bias) as well as the upper and lower limits of agreement are shown on each plot.

Mean value and standard deviation for all subjects for 1^st^ and 2^nd^ measurement can be seen in Table 1. As also seen in Fig. 4, mean values for the two sessions are very close, notably for the central measurements (sphere and cylinder).

**Table 1.** Central and peripheral mean Sphere (M) and Cylinder (C) and their standard deviation across all 15 subjects for 1^st^ and 2^nd^ measurement.

Session	Centre				Periphery			
	Mean M	STD M	Mean C	STD C	Mean M	STD M	Mean C	STD C
**1st**	−1.78	2.03	−1.47	0.78	−2.41	1.71	−3.43	1.36
**2nd**	−1.71	2.03	−1.41	0.78	−2.54	1.71	−3.32	1.37

The double pass instrument and its method for calculating astigmatism and defocus were found to be highly repeatable and accurate for measuring both central and peripheral refraction.

### 3.2 Comparison of the two setups

**Fig. 5.**
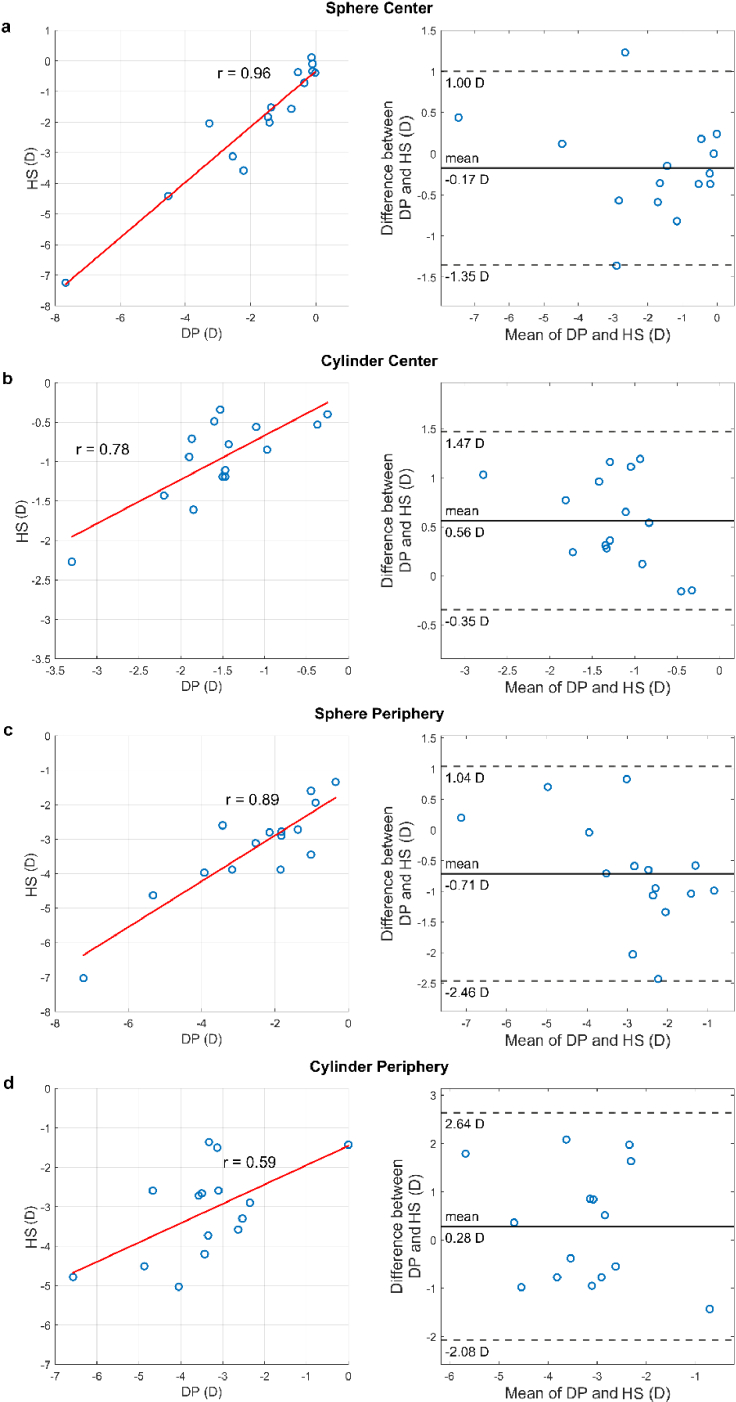
Scatter plot showing linear fit (least squares) and Pearson’s correlation coefficient (left) and Bland-Altman plot (right) for the central sphere (a), central cylinder (b), peripheral sphere (c) and peripheral cylinder (d) for 15 subjects using the two instruments.

The three-measurement mean sphere and cylinder values for all 15 subjects for both instruments at the two eccentricities can be seen in Table 2. The comparison between the measurement of the DP instrument and the laboratory prototype HS instrument can be seen in Fig. 5(a-d) where the correlation plots and the Bland-Altman plots between the measurement of each instrument are presented for central sphere (a), central cylinder (b), peripheral sphere (c) and peripheral cylinder (d).

**Table 2.** Central and peripheral mean Sphere (M) and Cylinder (C) for the two instruments for all 15 subjects.

	Centre				Periphery			
	DP M	HS M	DP C	HS C	DP M	HS M	DP C	HS C
**1**	−0.55	−0.37	−1.90	−0.94	−0.90	−1.94	−3.43	−4.20
**2**	−0.02	−0.39	−0.25	−0.40	−1.02	−1.60	−2.35	−2.90
**3**	−1.47	−1.83	−1.87	−0.71	−3.93	−3.97	−3.50	−2.66
**4**	−0.13	0.11	−1.53	−0.34	−0.35	−1.34	−3.35	−3.73
**5**	−4.53	−4.41	−1.85	−1.61	−5.33	−4.63	−4.67	−2.59
**6**	−1.42	−2.01	−1.10	−0.56	−1.83	−2.78	−3.10	−2.59
**7**	−0.10	−0.34	−0.37	−0.53	−1.83	−2.90	−4.05	−5.03
**8**	−0.75	−1.57	−1.50	−1.19	−2.15	−2.80	−3.13	−1.50
**9**	−0.35	−0.72	−3.30	−2.27	−1.02	−3.45	0.00	−1.43
**10**	−0.10	−0.10	−1.43	−0.78	−1.38	−2.72	−2.53	−3.30
**11**	−2.22	−3.58	−1.60	−0.49	−1.85	−3.88	−3.33	−1.36
**12**	−3.27	−2.04	−0.97	−0.85	−3.43	−2.60	−2.63	−3.58
**13**	−2.55	−3.12	−2.20	−1.43	−3.17	−3.88	−3.57	−2.72
**14**	−7.68	−7.24	−1.47	−1.19	−7.23	−7.03	−6.57	−4.78
**15**	−1.37	−1.52	−1.47	−1.11	−2.53	−3.12	−4.87	−4.51

## 4. Discussion

The double-pass principle has been used in the past to assess on-axis and off-axis image quality, by directly imaging the eye’s point spread function [17,20–22]. In this work we presented a double-pass based instrument for the assessment of central and peripheral refraction, using a tunable lens that allowed the fast acquisition of central and peripheral through focus images of the PSF. A novel image processing algorithm was used to analyse the images and compute defocus and astigmatism, without the need of trial lenses, making the measurement rapid and accurate. A total of 15 subjects were measured monocularly (right eye) while fixating binocularly at a far target. The instrument is based on the double-pass principle where a point source is projected onto the retina and its aerial image is captured using a camera. A common-path tunable lens is used to shift the focus of the point source, producing a sequence of through-focus double-pass images. Defocus and astigmatism are computed for two different retinal sites foveally and peripherally (30° nasal). The instrument’s repeatability was tested, and, subsequently, compared to defocus and astigmatism from a Hartmann-Shack aberrometer, under the same conditions such as light levels, fixation, and pupil size.

There was high repeatability at both angles and quantities measured; the DP measurement exhibited a mean difference of 0.06, 0.07D, 0.12D and 0.11D for central defocus, central astigmatism, peripheral defocus, and peripheral astigmatism respectively, suggesting that the measurement is more repeatable centrally than peripherally. This was to be expected, since in the peripheral measurement the pupil is seen as elliptical reducing the amount of light entering the pupil. In addition, high levels of astigmatism and higher order aberrations in the periphery lead to larger spot areas and can have an impact on the accuracy of the algorithm. Nevertheless, the low bias and limits of agreements in all four scenarios of Fig. 4 indicated a good overall repeatability of the DP instrument and the method.

Comparison between the two instruments (DP and HS) revealed two things: first, the methods showed both strong correlation and agreement in the measurement of defocus. This can be seen in Fig. 5(a-b) for central and peripheral defocus with correlation coefficients r = 0.96 ( $p=1.83\times 10^{-8}$ ) and r = 0.89 ( $p=1.04\times 10^{-5}$ ), respectively. Central defocus shows better correlation and agreement, and this can be attributed to the fact that in the peripheral measurement the pupil used for the two methods is not identical; the HS analysis method used here calculates the refraction from Zernike coefficients over a circular pupil [18,23], whereas the DP method measures image quality through the elliptical pupil, for pupils 3.5 mm or less.

Astigmatism, although highly correlated in the central measurement with a Pearson’s correlation coefficient of r = 0.78 ( $p=6.87\times 10^{-4}$ ), shows lower overall agreement than defocus and a larger number of outliers. This can be attributed partially to the fact that our DP algorithm for the calculation of astigmatism is based on the “ellipticity” of the image; an ellipse is fitted to the aerial image of the PSF and the ratio of the two axis is calculated for every through-focus image. The presence of asymmetric higher order aberrations, such a coma and second order coma, could contribute to an elliptical shape of the spot, even in the absence of astigmatism. This essentially means that the algorithm calculates the “effective” astigmatism of the subject, which is not identical with the astigmatism in the Zernike representation, expressed as the sum of the first three (2,4 and 6) astigmatism coefficients. Similarly, peripheral astigmatism shows lower agreement and correlation compared to defocus with a correlation coefficient of r = 0.59 ( p=0.02 ), which can be attributed to the latter effect. There might therefore be better agreement between DP and HS if an image plane metric had been used for HS instead, especially for peripheral refraction. Furthermore, as in the case of peripheral defocus, the difference in the actual pupil in the two measurements can further affect the disagreement.

Our peripheral refraction results are in-line with the literature [24]: peripheral astigmatism was found to be higher than central astigmatism for all except one subject, whose peripheral astigmatism was found to be lower for both measurement methods. For the DP method peripheral astigmatism varied between subjects from 0 D to −6.57 D with a mean value of −3.30 D and standard deviation 1.37 D. Smaller range of values were obtained using the HS method with subjects ranging from −1.36 D to −5.03 D, but with a similar mean value of 3.03 D and a standard deviation 1.12 D. Gustafsson et al, using aberrometry, they found that astigmatism at 30° of visual angle, varied between subjects from 1 to 7 D, with a mean astigmatism of about 4 D on the nasal side and about 1.5 D lower on the temporal side [25]. Similar values were found by Seidemann et al [26] using photorefraction and the double-pass technique, and Atchison et al [27] using aberrometry.

There are several tools to assess retinal image quality; the HS wavefront sensor is one of the most used ones. The HS wavefront aberrometers are commonly used because they are fast and accurate, however, they can misestimate retinal image quality in eyes higher intraocular scattering, as shown in the study by Diaz-Douton et al [28]. In the DP method the retinal image information is assessed directly, and therefore, intraocular scattering and higher-order aberrations do not affect the measurement. Furthermore, recent developments in camera sensors technology, allows for much higher resolution, sensitivity, speed and overall form factor at a fraction of a price and have significantly improved the robustness of the double pass technique, while reducing the overall cost.

Finally, it is important to note that, although, we chose only two eccentricities (0° and 30° nasal), the DP instrument can measure refraction, practically at any angle. Future development of the instrument involves upgrades to facilitate measurement at more eccentricities in an automated fashion, which will significantly decrease measurement time while increasing peripheral data.

## 5. Conclusion

A double-pass instrument for the in-vivo assessment of central and peripheral refraction was developed and tested on a set of 15 healthy subjects. The instrument and the corresponding method for calculating central and peripheral refraction was compared against an HS laboratory prototype and found to have good correlation and agreement, particularly for the measurement of defocus. The instrument can become a useful research and clinical tool in myopia management and also in the assessment of peripheral image quality in pseudophakic patients.

## Data Availability

Data of this paper are not publicly available, but it may be obtained from the corresponding author upon reasonable request.
